# Is Interpersonal Trust or Institutional Trust More Strongly Associated with Subjective Well-Being than Mental Health Among Financially Insecure U.S. Southern Households?

**DOI:** 10.3390/healthcare14040485

**Published:** 2026-02-14

**Authors:** Shari C. Moxley, Charleen C. McNeill, M. E. Betsy Garrison

**Affiliations:** 1 School of Human Environmental Sciences, University of Arkansas, Fayetteville, AR 72701, USA; moxley@uark.edu; 2 College of Nursing and Health Professions, The University of Southern Mississippi, Hattiesburg, MS 39406, USA; charleen.mcneill@usm.edu

**Keywords:** social capital, depression, anxiety, quality of life, healthcare policy, patient care, financial insecurity, vulnerable populations, survey research

## Abstract

**Background:** Subjective well-being (SWB) is an important outcome in healthcare. Identifying correlates of SWB among economically vulnerable populations can inform healthcare delivery and policy decisions. **Objective:** This study examines whether social capital is more strongly associated with evaluative SWB than mental health among financially insecure households in the U.S. South. **Methods:** Data were drawn from the SR-Stat Baseline Survey 2024. Multiple regression analysis was used to examine the relative associations of interpersonal trust, institutional trust, and mental health with evaluative SWB, while controlling for socioeconomic-demographic characteristics. **Results**: Interpersonal trust, institutional trust, and mental health were each significantly associated with evaluative SWB. Institutional trust exhibited the largest standardized association, followed closely by mental health and interpersonal trust, with differences in magnitude remaining modest across variables. Several control variables, particularly age, income, and health insurance coverage, were also independently associated with SWB. Healthcare systems and policymakers may benefit from incorporating trust-building practices, such as transparent communication and equitable service delivery, alongside mental health screening and support, to address multiple dimensions of well-being in economically vulnerable populations. **Conclusions:** Evaluative SWB among financially insecure households is associated with mental health and trust-based social capital at both interpersonal and institutional levels. These findings highlight the importance of addressing individual functioning and broader institutional contexts when designing interventions aimed at improving well-being.

## 1. Introduction

Subjective well-being (SWB) is a central outcome in public health and population research, yet it encompasses distinct components, including evaluative (cognitive) judgments about one’s life circumstances and affective (experiential) states such as happiness, stress, and sadness [1,2,3]. In the present study, SWB is conceptualized specifically as evaluative SWB, defined as individuals’ satisfaction with concrete living conditions and opportunities in their communities. This focus on evaluative SWB reflects how people cognitively assess their quality of life rather than momentary emotional states and aligns directly with the study’s operationalization, which is based on satisfaction across multiple life domains [3].

Understanding factors associated with evaluative SWB is particularly important amid rising economic insecurity and institutional strain, conditions that shape how individuals judge their circumstances and future prospects [3,4,5]. Two factors that are frequently examined in relation to SWB, mental health and social capital, operate through conceptually distinct pathways. Mental health represents an individual-level determinant of SWB. Symptoms of depression, anxiety, and loneliness directly impair emotional functioning, daily activities, and social relationships, leading to lower evaluations of life satisfaction and quality of life [1,6]. Extensive longitudinal and clinical evidence demonstrates that poorer mental health is consistently associated with lower SWB across populations, with changes in depressive symptoms closely tracking changes in life satisfaction [6,7]. Financial insecurity further intensifies this relationship by increasing psychological distress and perceived stress, particularly among low-income households [2,4,5,7,8].

Social capital, in contrast, reflects contextual and relational resources embedded in social systems. Social capital theory emphasizes that trust, norms of reciprocity, and shared expectations facilitate cooperation and access to resources that support individual and collective well-being [3,9,10]. Within this framework, cognitive social capital, expressed through trust, is analytically distinct from structural social capital, such as civic participation [3,10]. Trust-based social capital has been linked to improved health outcomes, resilience, and SWB across diverse populations [2,7,11,12]. Cognitive social capital itself comprises multiple dimensions. Interpersonal trust refers to expectations of reliability and goodwill in everyday interactions with others, including neighbors, coworkers, educators, healthcare providers, and service workers. Institutional trust reflects confidence in the fairness, competence, and integrity of formal institutions such as local government, police, and media [13,14]. These dimensions correspond to different levels of influence; interpersonal trust operates primarily at the relational or meso level, whereas institutional trust reflects broader systemic and contextual conditions [2,3,11]. Both have been associated with SWB, although evidence suggests that their strength and direction may vary by socioeconomic context [2,7,10,14].

Comparing mental health with interpersonal and institutional trust is theoretically justified because each construct captures distinct mechanisms shaping evaluative SWB. Mental health reflects internal psychological functioning, interpersonal trust reflects access to informal support and social reciprocity, and institutional trust reflects perceived fairness, effectiveness, and accessibility of systems that structure daily life [2,3,12,15]. In economically vulnerable populations, institutional trust may be particularly salient, as confidence in public systems influences expectations regarding resource access, protection, and social inclusion [3,15]. At the same time, interpersonal trust may buffer stress by facilitating informal coping and support, while mental health remains a proximal determinant of well-being [1,6,11,16].

What is missing from the existing literature is not evidence that mental health or trust relate to SWB but rather a comparative analysis of their relative associations within contexts of economic and institutional vulnerability. Prior studies have often examined these factors separately or within general populations despite evidence that financial insecurity alters both psychological processes and the role of trust in shaping well-being [4,5,17,18]. Financially insecure households experience heightened stress, reduced access to resources, and greater exposure to institutional barriers, conditions that may further amplify the importance of both interpersonal and institutional trust [3,4,16].

Accordingly, the present study addresses this contextual gap by examining the relative associations of mental health, interpersonal trust, and institutional trust with evaluative SWB among financially insecure households in the U.S. South, while controlling for key socioeconomic-demographic characteristics (SDC). Using data from the SR-Stat: Baseline Survey 2024, this analysis adopts a comparative modeling approach to assess whether these individual- and system-level factors exhibit similar or differing relationships with SWB in a setting characterized by economic strain and institutional reliance [2,3,16]. Given the cross-sectional design, the study does not assume causal directionality but instead evaluates the magnitude and complementarity of associations, providing a foundation for future longitudinal research.

### Review of Literature

Subjective well-being (SWB) is widely recognized as a multidimensional construct encompassing both evaluative judgments and affective experiences [19,20]. This distinction is well established in the well-being literature and is reflected in international measurement guidance, which emphasizes that life evaluation captures stable judgments that are sensitive to socioeconomic and institutional context [21]. Focusing on evaluative SWB is therefore appropriate for examining how individual and contextual factors shape perceived quality of life in financially insecure populations.

Mental health represents a proximal, individual-level determinant of evaluative SWB. Extensive evidence indicates that symptoms of depression, anxiety, and loneliness impair daily functioning, social relationships, and life satisfaction, leading to lower evaluations of quality of life [1,6]. Longitudinal research shows that declines in mental health typically precede or accompany declines in SWB, while improvements in mental health are associated with subsequent gains in life satisfaction [1]. Financial insecurity further exacerbates these relationships, as economic strain and perceived instability are strongly associated with psychological distress and depressive symptoms, particularly among low-income households; as a result, mental health is an essential comparator when examining other factors of evaluative SWB in economically vulnerable contexts [4,5].

In contrast, social capital, particularly its cognitive dimension of trust, reflects relational and systemic resources that shape how individuals evaluate their lives through expectations of reciprocity, fairness, and access to support [3,9]. Cognitive social capital is analytically distinct from structural social capital, such as civic participation, and has been shown to relate more consistently to well-being outcomes [9,10]. Within this domain, researchers commonly distinguish between interpersonal trust, referring to confidence in others encountered in everyday social interactions, and institutional trust, referring to confidence in formal institutions such as local government, police, and media [14,15]. These forms of trust operate at different levels, with interpersonal trust reflecting relational environments and institutional trust reflecting broader systemic conditions.

A growing body of empirical research links both interpersonal and institutional trust to SWB, although the strength and direction of these associations vary by context. Meta-analytic evidence demonstrates positive associations between trust and SWB across the life course, with evidence of bidirectional reinforcement over time [2]. Cross national studies further suggest that social capital, particularly trust, is more strongly associated with SWB in low income or institutionally weaker contexts than in high income settings [7]. At the same time, country-level panel analyses indicate that causal directionality between trust and SWB may be modest or context-dependent, especially for institutional trust [12]. These findings underscore the need for careful, non-confirmatory modeling approaches.

The relevance of trust for SWB appears particularly salient under conditions of economic and institutional vulnerability. Among financially constrained populations, institutional trust may shape expectations regarding access to public services, protection from risk, and fairness in resource distribution, all of which influence evaluations of life circumstances [3,16]. Interpersonal trust, in turn, may buffer the psychological effects of financial stress by facilitating informal support and collective coping [17]. However, financial strain remains a well-documented risk factor for depression and diminished quality of life, highlighting the importance of examining trust and mental health jointly rather than in isolation [4,8].

Age, gender, race, and educational attainment are foundational drivers of SWB, as well as mental health and social capital. Life course research shows that evaluative SWB varies systematically with age, often following nonlinear patterns linked to changing health, social roles, and economic security [19,20]. Gender differences in SWB and mental health are well documented, with women reporting higher rates of anxiety and depressive symptoms, particularly under conditions of financial stress, which may translate into lower evaluations of quality of life [6,8]. Race and ethnicity further shape exposure to structural stressors, discrimination, and institutional barriers, all of which influence trust in institutions and access to supportive resources, making racial and ethnic identity salient factors related to both SWB and trust-based social capital [3,7]. Educational attainment is similarly central, as it is associated with income stability, health literacy, civic engagement, and institutional navigation, each of which may affect both evaluative SWB and levels of interpersonal and institutional trust [3,20].

Economic and family-related characteristics, including income, marital status, employment status, and health insurance coverage, warrant inclusion given their direct and indirect links to well-being, mental health, and trust in systems. Income remains one of the most robust correlates of life satisfaction, even when financial insecurity is measured subjectively, reflecting both material conditions and perceived economic vulnerability [20,21]. Marital status is associated with SWB through emotional support, resource pooling, and social integration, while job loss or unstable employment is a well-established risk factor for psychological distress and diminished quality of life [4,19]. Health insurance coverage is particularly salient in financially insecure populations, as it affects access to care, perceived protection from financial risk, and confidence in healthcare systems, thereby intersecting with both mental health and institutional trust [22,23,24]. Including these SDC as controls is therefore essential to isolate the relative associations of mental health, interpersonal trust, and institutional trust with evaluative SWB and to avoid attributing contextual or structural effects to individual-level or relational constructs.

Although prior research has established associations between trust, mental health, and SWB, relatively few studies have directly compared these factors within populations experiencing sustained economic and institutional vulnerability. The existing evidence further suggests that the strength and direction of trust’s associations with SWB are context-dependent, with mixed findings regarding causal ordering. Taken together, the literature indicates that mental health, interpersonal trust, and institutional trust are each linked to evaluative SWB, yet these factors are rarely examined comparatively in economically vulnerable populations. A comparative modeling approach that examines interpersonal and institutional trust alongside mental health among financially insecure households is therefore warranted, as it enables assessment of the relative magnitude and complementarity of individual-level and contextual correlates of evaluative well-being without presuming causal direction in cross-sectional data.

## 2. Materials and Methods

### 2.1. Dataset

Data from the SR-Stat: Baseline Survey 2025, developed by the North Central Regional Center for Rural Development, the Southern Rural Development Center, and Auburn University and funded by the National Institute of Food and Agriculture were used in the current study [25]. This recently released dataset, which included quota sampling nested within states derived from U.S. Census Bureau resources, enables timely analysis among a large, regionally representative sample of adults in the southern United States. Following the Institutional Review Board approval from Purdue University (IRB number: 2023-1420), data were collected in 2024 using a Qualtrics XM survey, in which the first question secured informed consent, administered to residents across 13 United States Department of Agriculture southern states. Of the 5500 respondents, 3472 were financially insecure in that they responded they had trouble keeping up with their household bills for at least 6 out of the last 12 months and comprised the sample of the current study. The sample was limited to financially insecure households because sustained economic strain is a key contextual factor shaping mental health, trust in others and institutions, and evaluations of quality of life, and prior research indicates that both psychological distress and the salience of trust-based resources and institutional confidence vary systematically under conditions of financial insecurity [4,16,22].

The respondents ranged in age from 18 to 92 years (M = 50, SD = 16.4), 52% were women; 64% White and 22% Black; 39% had earned a high school diploma or equivalent; 51% were married or living with a partner; 39% work full-time; and 60% reported annual household incomes below $50,000. A little over 25% had their health insurance through their own or another family member’s current or previous employer. About the same percentage either did not have health insurance (11%) or purchased it directly from an insurance company (12%). Almost 20% reported having problems paying their family’s medical bills.

### 2.2. Variables

Subjective Well-Being: Sixteen cognitive-focused questions about satisfaction with various aspects of quality of life were used to measure SWB. These aspects included questions about availability, access, and opportunities within a respondent’s community. The response set was a five-point Likert scale from ‘very unsatisfied’ to ‘very satisfied’, with ‘neutral’ as the middle category. Because an exploratory factor analysis revealed a single factor with a very high eigenvalue (8.14), 50% of the variance and a KMO of 0.95, and only one other factor with an eigenvalue over 1.0, a confirmatory factor analysis was conducted with a single forced factor. Factor loadings ranged from 0.60 (pollution levels) to 0.78 (employment opportunities), and a single variable was computed by saving the factor loadings for the regression analysis. Factor scores were used rather than a summed scale to account for differential item loadings and to reduce measurement error in the composite SWB construct. Higher scores indicate a greater quality of life.

Mental Health: The data set contained four questions about mental health that captured general mental health functioning rather than specific mental health disorders, consistent with population-level surveys. These questions were having little interest in doing things; feeling down, depressed, or hopeless; feeling anxious, nervous, or on edge; and feeling lonely. For the first three items, respondents were directed to consider how often they have been bothered by these problems over the last 3 months. The four-point response set ranged from nearly every day (1) to not at all (4). For the loneliness item, respondents were asked how often they felt lonely with a five-point response set ranging from often/always (1) to never (5). Because of the differences in the two response sets, variables were standardized and then summed into a composite score. Mental health items were coded such that higher values reflect fewer symptoms and better mental health.

Interpersonal trust: Six items were summed to measure interpersonal trust, including people: in your neighborhood, you work with; at your church or place of worship; who work in the stores where you shop; who educate your children; and who provide your health care services. Respondents were directed to consider how much they trust people in their communities. The response set ranged from strongly distrust (1) to strongly trust (5), with neither trust nor distrust as the middle category. Higher scores indicate a higher level of trust.

Institutional Trust: Three items were summed to measure institutional trust, including local government, local news and media, and the police in your local community. Respondents were directed to consider how much they trust institutions in their communities. The response set ranged from strongly distrust (1) to strongly trust (5), with neither trust nor distrust as the middle category. Higher scores indicate higher level of trust.

Socioeconomic-demographic characteristics (SDC): Eight items measured SDC by standard survey questions, including age, gender, race/ethnicity, marital status, educational attainment, employment status, household income, and health insurance coverage. These variables included the regression analysis as control variables, and categorical variables were dummy coded. The reference categories were ‘women’ for gender, ‘self-identified White’ for race/ethnicity, ‘married or coupled’ for marital status, ‘employed full-time’ for employment status, and ‘yes, having any of kind of health insurance’ for health insurance coverage.

### 2.3. Data Analysis

Data were exported into SPSS (version 29). Following univariate, including reliability analyses, and bi-variable (first-order correlations) analyses, a multiple regression analysis was conducted to address the objective. Multiple regression analysis was chosen because of the continuous independent and dependent variables. Initially, the control SDC variables, dummy coded where necessary, were regressed on the dependent variable, SWB. The independent variables of interpersonal and institutional trust and mental health were then added to the model.

## 3. Results

The means of the 16 items of the individual SWB variables ranged from 3.42 (SD = 1.10), ‘Access to affordable housing,’ to 4.10, ‘availability of internet,’ (SD = 0.96). The Cronbach’s alpha was 0.93. As indicated previously, factor scores were computed, saved, and entered into the regression analyses.

Prior to standardizing, the means of the four items of the mental health variables ranged from 3.15 ‘feeling anxious, nervous, or on the edge’ (SD = 1.00) to 3.23 ‘feeling down, depressed or hopeless’ (SD = 0.97) and the mean of feeling lonely was 3.31 (SD = 1.35). The scale mean of the four items was 9.58 (SD = 0.89), and the Cronbach’s alpha was 0.89.

The means of the six items of interpersonal trust ranged from 3.57 ‘people who work in the stores where you shop’ (SD = 0.87) to 4.01 ‘people who provide your health care services’ (SD = 0.91. The scale mean was 22.31 (SD = 4.57), and the Cronbach’s alpha was 0.86.

The means of the three items of institutional trust ranged from 3.14 ‘local government’ (SD = 1.11) to 3.53 ‘police in your local community’ (SD = 1.13. The scale mean was 9.58 (SD = 2.68), and the Cronbach’s alpha was 0.78.

The correlations between the eight control variables were all in the expected direction and ranged from 0.02 (marital status and age) to 0.43 (income and education). The correlations between the control variables and the independent variables ranged from 0.02 (employment status and institutional trust) to 0.35 (employment status and interpersonal trust). The correlations between the control variables and dependent variables ranged from −0.01 (race/ethnicity) too 0.21 (income). The correlation between the independent variables ranged from 0.05 (interpersonal trust and mental health) to 0.35 institutional trust and mental health). The correlations between the independent variables and the dependent variable ranged from 0.37 (interpersonal trust) to 0.45 (institutional trust).

The results of the first regression analysis indicated that the model was statistically significant and revealed that seven of the eight control variables were significantly related in the expected direction to SWB (Table 1).

Age had the strongest association with SWB, followed by income, even in a study of financial insecure households. Being employed was not found to be significantly associated with SWB.

The results of the second regression analysis indicated that the model was statistically significant, with an associated significant change in R^2^. Three of the eight control variables were found to be significantly associated in the expected direction with SWB, namely age, income, and having health insurance. Multicollinearity diagnostics indicated no substantial concern, as the variance inflation factors (between 1.05 and 1.62) were well within established thresholds. All three independent variables were found to be significantly related to SWB, with institutional trust having a slightly larger standard association than interpersonal trust or mental health (β = 0.27 compared with 0.26 or 0.25). These results indicate the complementary nature of interpersonal trust, mental health, and institutional trust and suggest that institutional trust, in particular, may warrant further attention in financially insecure households.

## 4. Discussion

The present study examined whether interpersonal trust and institutional trust, as dimensions of cognitive social capital, are associated with variation in evaluative SWB more strongly than mental health among financially insecure households in the U.S. South. Consistent with prior research on social determinants of health and well-being, all three variables were significantly associated with SWB after controlling for a set of SDC [3,19,20]. These findings reinforce evidence that mental health, interpersonal trust, and institutional trust each represent distinct yet meaningful correlates of evaluative well-being in economically vulnerable populations [2,4].

Although institutional trust exhibited the strongest association with SWB in the fully adjusted model, the magnitude of the coefficients across the three variables was similar, indicating a complementary pattern rather than a strict hierarchy of influence. Importantly, the similarity in coefficient magnitudes suggests that these constructs function in complementary rather than hierarchical ways. Prior meta-analytic and longitudinal research suggests that trust and well-being may reinforce one another over time, with variation in strength depending on socioeconomic and institutional context [2,12]. As such, the observed differences in coefficient size should be interpreted cautiously and should not be understood as diminishing the importance of mental health or interpersonal trust, both of which retained statistically and substantively meaningful associations with SWB [1,17].

The salience of institutional trust may be particularly pronounced in financially insecure households, where dependence on public systems and formal services is elevated. Prior studies indicate that institutional trust is closely linked to perceptions of fairness, access to resources, and confidence in governance and service delivery, all of which shape evaluations of quality of life under conditions of constraint [3,16]. In this context, institutional trust may function as an indicator of perceived system responsiveness that influences SWB independently of individual mental health status [7].

At the same time, interpersonal trust reflects the quality of everyday social relationships that can facilitate informal support, social cohesion, and shared coping, which are especially important under economic strain [9,17]. These relational resources may buffer stress and contribute to well-being even when material circumstances are constrained, supporting conceptual distinctions between interpersonal and institutional trust while underscoring their joint relevance [10].

Mental health demonstrated a robust association with SWB, consistent with extensive evidence linking depressive and anxiety symptoms to diminished life satisfaction and quality of life [1,6]. Rather than viewing mental health and trust as competing explanations, the findings align with research suggesting that psychological functioning and social context jointly shape how individuals evaluate their lives, particularly in economically insecure environments [4,22]. This interpretation is reinforced by the fact that several SDC, particularly age, income, and health insurance coverage, remained independently associated with SWB, highlighting the layered and interacting determinants of well-being in financially insecure populations [19,23].

From a healthcare and policy perspective, these results suggest that efforts to enhance SWB among financially insecure households may benefit from integrated approaches that address both mental health needs and trust-based contextual factors. Mental health screening and treatment remain essential, given the elevated prevalence of psychological distress associated with financial strain [4,8]. In parallel, policies and practices that foster institutional trust, including transparent communication, equitable service delivery, and culturally responsive care, may improve engagement with healthcare systems and influence perceptions of care quality and access [3,16].

The broader context of financial insecurity is critical to interpretation. Economic strain is associated with higher rates of depression, lower access to healthcare, and increased exposure to institutional barriers, all of which are linked to reduced SWB [22,23]. Rising healthcare costs and gaps in insurance coverage may further erode institutional trust, particularly when unmet needs are experienced as indicators of unfairness or systemic neglect rather than solely individual hardship [16,24]. Institutional trust in this setting may thus reflect cumulative experiences with affordability, access, and perceived legitimacy of healthcare systems.

Several limitations should be acknowledged. The cross-sectional design limits causal inference, and the observed associations cannot determine temporal ordering or directionality [12]. In addition, differences in scale length across trust measures and the absence of statistical tests comparing coefficient magnitudes among the independent variables should be considered when interpreting relative associations. All measures were self-reported and collected via an online survey, which may introduce recall or response bias and limit participation among individuals with constrained internet access. Additionally, interpersonal trust was measured using more items than institutional trust, which may affect scale reliability and variance, and mental health and social capital were operationalized using conceptually distinct instruments, which may influence comparability across factors [10]. Moreover, mental health was assessed using a brief four-item composite derived from survey items capturing symptoms of depression, anxiety, and loneliness, rather than a comprehensive diagnostic or screening instrument, which allowed the study to reflect general mental health status while minimizing respondent burden and survey fatigue. Finally, the findings pertain specifically to financially insecure households in the U.S. South and should not be generalized to economically secure populations.

Despite these limitations, this study extends the existing literature by providing a context-specific comparative analysis of mental health, interpersonal trust, and institutional trust in relation to evaluative SWB. By focusing on financially insecure households, the results highlight how individual-level psychological factors and broader relational and institutional contexts jointly contribute to perceptions of quality of life. Future research would benefit from longitudinal designs, investigation of mediating pathways such as perceived stress and healthcare utilization, and comparative analyses across regions and policy environments to further clarify these relationships [2,5].

### Implications

The findings suggest several practice-relevant implications for healthcare systems and related public institutions serving financially insecure populations. Institutional trust was slightly more strongly associated with evaluative SWB than either interpersonal trust or mental health, even after accounting for SDC. This pattern indicates that perceptions of institutional fairness, transparency, and reliability may shape how economically vulnerable households evaluate their quality of life in ways. For healthcare systems, this underscores the importance of trust-building efforts that operate at the institutional level, such as transparent communication about eligibility, costs, and service availability, consistent enforcement of policies, and visible coordination with trusted local institutions. Incorporating measures of institutional trust into community health needs assessments, patient experience surveys, or quality improvement frameworks may help healthcare organizations better understand how broader system perceptions intersect with patient well-being.

The results also point to opportunities for integrating mental health screening with trust-oriented interventions rather than treating these domains as separate. Although mental health remained a significant correlate of SWB, its association was comparable in magnitude to both interpersonal and institutional trust, suggesting that interventions focused solely on symptom reduction may overlook important contextual influences. For example, mental health screening initiatives in primary care or community settings could be paired with referral pathways that emphasize institutional navigation support, such as assistance with insurance enrollment, benefits access, or connections to local government and community services. At the policy level, efforts to reduce administrative complexity, improve communication across healthcare and social service systems, and address perceived inequities in access may indirectly support mental health and SWB by strengthening institutional trust. Importantly, these implications are grounded in observed associations rather than causal claims and highlight how healthcare and policy interventions can attend simultaneously to individual-level mental health needs and broader institutional conditions that shape well-being among financially insecure households.

## 5. Conclusions

This study contributes to the literature by providing a context-specific, comparative analysis of key correlates of evaluative SWB within a population for whom economic insecurity is a defining condition. Findings indicate that mental health, interpersonal trust, and institutional trust are each meaningfully associated with evaluative SWB among financially insecure households in the U.S. South, even after accounting for a broad set of SDC. Although institutional trust exhibited the largest standardized association in the comparative models, the differences in magnitude across the three independent variables were modest, underscoring the complementary roles of individual-level psychological functioning and broader relational and institutional contexts in shaping evaluations of quality of life.

These findings have important implications for healthcare systems and policy. Efforts to improve well-being among financially insecure households should not focus exclusively on individual mental health needs but should also address trust-related dimensions of the healthcare and institutional environment. Transparent communication, equitable service delivery, culturally responsive care, and reduced administrative barriers may help strengthen institutional trust, while community-based initiatives and integrated care models can support interpersonal trust and mental health simultaneously.

## Figures and Tables

**Table 1:** Multiple regression analysis of socioeconomic-demographic characteristics, trust, and mental health predicting subjective well-being.

Variable	Model 1		Model 2	
	β	*t*	β	*t*
Age	0.17	7.64 ***	0.09	4.63 *
Education	0.06	2.91 **	0.02	0.87
Income ^r^	0.11	5.03 ***	0.06	3.11 **
Female ^r^	−0.07	−3.63 ***	−0.02	−1.18
Self-Identified as White ^r^	−0.09	−4.44 ***	−0.03	−1.88
Coupled ^r^	0.09	4.48 ***	0.01	0.78
Employed ^r^	0.04	1.88	−0.03	−1.43
Has Health Insurance ^r^	0.10	5.11 ***	0.05	3.12 **
Interpersonal Trust			0.26	13.88 ***
Institutional Trust			0.27	14.95 ***
Mental Health			0.25	14.28 ***
R^2^	0.31		0.59	
Adj. R^2^	0.10		0.35	
ΔR^2^			0.25 ***	

Note. ^r^ = Reference category. Overall fit: model 1, F(_8, 34,723_) = 34.37, *p* < 0.001; model 2, F(_11, 3720_) = 72.99, *p* < 0.001. *p* < 0.05 *, *p* < 0.01 **, *p* < 0.001 ***.

## Data Availability

The data are publicly available from Southern Region Household Data. SR-Stat: Baseline Survey 2024, https://purr.purdue.edu/publications/4794/1 (accessed on 11 February 2026).

## Abbreviations

The following abbreviations are used in this manuscript:

SDC	socioeconomic-demographic characteristics
SWB	subjective well-being
